# Neuroanatomical Alterations in Patients with Early Stage of Unilateral Pulsatile Tinnitus: A Voxel-Based Morphometry Study

**DOI:** 10.1155/2018/4756471

**Published:** 2018-02-28

**Authors:** Yawen Liu, Han Lv, Pengfei Zhao, Zhaohui Liu, Wenjing Chen, Shusheng Gong, Zhenchang Wang, Jian-Ming Zhu

**Affiliations:** ^1^Institute for Biomedical Engineering and College of Information Engineering, China Jiliang University, Hangzhou, China; ^2^Department of Radiology, Beijing Friendship Hospital, Capital Medical University, Beijing, China; ^3^Department of Radiology, Beijing Tongren Hospital, Capital Medical University, Beijing, China; ^4^Department of Otolaryngology Head and Neck Surgery, Beijing Friendship Hospital, Capital Medical University, Beijing, China

## Abstract

During the past several years, the rapid development of neuroimaging techniques has contributed greatly in the noninvasive imaging studies of tinnitus. The aim of the present study was to explore the brain anatomical alterations in patients with right-sided unilateral pulsatile tinnitus (PT) in the early stage of PT symptom using voxel-based morphometry (VBM) analysis. Twenty-four patients with right-sided pulsatile tinnitus and 24 age- and gender-matched normal controls were recruited to this study. Structural image data preprocessing was performed using VBM8 toolbox. Tinnitus Handicap Inventory (THI) score was acquired in the tinnitus group to assess the severity of tinnitus and tinnitus-related distress. Two-sample *t*-test and Pearson's correlation analysis were used in statistical analysis. Patients with unilateral pulsatile tinnitus had significantly increased gray matter (GM) volume in bilateral superior temporal gyrus compared with the normal controls. However, the left cerebellum posterior lobe, left frontal superior orbital lobe (gyrus rectus), right middle occipital gyrus (MOG), and bilateral putamen showed significantly decreased brain volumes. This was the first study which demonstrated the features of neuroanatomical changes in patients with unilateral PT during their early stages of the symptom.

## 1. Introduction

Tinnitus is an abnormal perception of sound in the absence of any external stimulus. Up to 15% of people in the world suffer from tinnitus [1, 2]. Tinnitus can be divided into nonpulsatile tinnitus (NPT) and pulsatile tinnitus (PT). Most patients suffer from NPT. Less than 10% of tinnitus patients are classified as PT subtype [3, 4]. Tinnitus is a disorder much more than just sound sensation. Patients who suffered from persistent tinnitus often accompanied with insomnia, anxiety, and other tinnitus-associated distress and abnormality [5]. The quality of life of tinnitus patients is seriously affected.

Unlike NPT, the rhythm of PT is always present in patients as water flow, wind blowing, or beat of the drum in their ears coinciding with the patients' heartbeats. Usually, the abnormal sounds could be significantly suppressed by compressing the internal jugular vein on the symptomatic side (if it is venous in origin; other origins may include dural arteriovenous shunts, paraganglioma, and involuntary contraction of muscles in the middle ear [6–10]). According to previous studies, focal sigmoid plate dehiscence (SPD) due to focal bone defects in the region of the sigmoid sinus is one of the very common etiologies of PT [3, 4, 11–14], which accounts for about 43%~60% populations in patients with PT [3, 14–16]. The abnormal sounds from vascular blood flow could be acquired by the inner ear through SPD. In this regard, CT arteriography and venography (CTA/V) is the most useful imaging method to pinpoint the etiology of PT [6, 16, 17].

During the past several years, the rapid development of neuroimaging techniques has contributed greatly in the noninvasive imaging studies of tinnitus. Various studies have shown that if functional changes occur, structural changes are likely found in patients with NPT as well [18–20]. As indicated by the previous studies, changes in neuroanatomical, especially volumetric changes in gray matters (GM), were important findings in NPT patient population [18, 21–25]. PT, as a subtype of tinnitus, was also proved to be a kind of symptom featured by neurofunctional alterations using resting-state functional magnetic resonance imaging (rs-fMRI) technique according to previous studies [26–31]. Generally, tinnitus with duration of up to 6 months is considered to be in the acute stage. But for rs-fMRI studies on tinnitus, previous studies defined that duration less than 48 months is the recent onset of disease [32, 33]. For studies on PT patients, researchers also detected neurofunctional changes in PT patients with disease duration less than 48 months [26–29, 31]. Thus, we defined those subjects with duration less than 48 months as PT patients in the early stage of the symptom. However, none of those studies reported neuroanatomical changes of PT patients. As a result, the neuroanatomical changes in PT patients still remain unclear.

Various morphological research techniques, such as voxel-based morphometry (VBM), surface-based morphometry (SBM), and deformation-based morphometry (DBM), could be used to explore neuroanatomical changes in tinnitus patients. Among these methods, VBM is one of the neuroimaging analysis techniques that allow investigation of focal differences in brain anatomy by using statistical parametric mapping approach. It had been widely used in many neuroimaging studies, especially researches in NPT patients [18, 21–24]. When we specifically analyze neuroanatomical alteration features in PT patients in this study, VBM analytic method would also be a candidate approach to make the results comparable with that of previous studies.

In this study, we aim to explore the brain anatomical alterations in patients with right-sided unilateral pulsatile tinnitus, which is much more common in the clinic. Our study will be an important addition to the neuroimaging research on patients with tinnitus, especially patients with right-sided pulsatile tinnitus.

## 2. Materials and Methods

### 2.1. Subjects

This study was approved by the medical research ethics committees and institutional review board. Written informed consent forms were obtained from each subject.

Since the limited population of pulsatile tinnitus patients, part of the patients in this study was collected from previous studies according to the same inclusion and exclusion criteria from Beijing Tongren Hospital; we also included patients and normal controls with current institutions including Beijing Friendship Hospital. In total, twenty-four patients with right-sided pulsatile tinnitus were recruited to this study. The patient inclusion criteria were set as follows: (1) right-sided unilateral pulsatile tinnitus with typical clinical symptom; (2) symptom duration is less than 48 months (defined as the early stage of PT symptom according to previous studies [26–29]); and (3) the etiology could be confirmed by CTA/V examination as SPD on the right side [6, 14, 16, 17, 34]. The exclusion criteria included (1) patients with significant hearing loss (hearing thresholds <25 dB HL at examined frequencies ranging from 250 Hz to 8 kHz in the pure-tone audiometry examination); (2) patients diagnosed with any other types of tinnitus, especially NPT; (3) patients with hyperacusis; (4) patients with neurological diseases, such as dementia and Alzheimer's disease; (5) patients with any kind of otologic conditions such as Meniere's disease and otosclerosis; and (6) patients who contraindicate with MRI examination. Tinnitus Handicap Inventory (THI) scores were also acquired in the patient group to assess the severity of disease and tinnitus-related distress [33, 35, 36]. All of the patients were recruited in the clinic. The intensity of pulsatile tinnitus was not measured since there was no standard in the clinic. Twenty-four age-, gender-, and handedness-matched healthy volunteers were also enrolled as normal controls. Exclusion criteria for normal healthy volunteers were the same as above. The characteristics of the subjects are presented in Table 1.

### 2.2. MRI Scanning

A 3 T GE Signa Excite MRI scanner (General Electric Medical Systems, Milwaukee, WI, USA), equipped with an eight-channel phased-array head coil, was used to collect structural brain images. All imaging studies were performed at the Medical Imaging Research Center of Beijing Friendship Hospital. Parallel imaging was employed in data acquisition. High-resolution 3D structural images were acquired with a 3D-BRAVO pulse sequence with the following acquisition parameters: TR (repetition time) = 8.8 milliseconds (ms), TE (echo time) = 3.5 ms, TI (inversion time) = 450 ms, field of view (FOV) = 24 cm × 24 cm, matrix = 256 × 256, and slice thickness = 1 mm without gap. In total, 196 slices of images were obtained from each subject.

### 2.3. Image and Data Processing

Image data preprocessing was performed using VBM8 toolbox in SPM8 software package (Statistical Parametric Mapping, Wellcome Department of Cognitive Neurology, London, UK) running under MATLAB. VBM8 toolbox provides a set of preprocessing modules including spatial normalization, segmentation, and other steps [37]. First, each subject's image set was spatially normalized to the Montreal Neurological Institute (MNI) 152 template using high-dimensional DARTEL algorithm [38]. The spatially normalized images were then segmented into gray matter (GM), white matter (WM), and cerebrospinal fluid (CSF). Modulation was also applied in order to avoid volumetric deformation of the GM due to stretching and shrinking effects during the normalization procedure. Next, the modulated GM images were smoothed with a 6 mm full width at half maximum (FWHM) isotropic Gaussian kernel. Finally, the smoothed GM images were resampled to a 3 mm × 3 mm × 3 mm voxel size for the statistical analysis.

### 2.4. Statistical Analysis

A two-sample two-tailed *t*-test was used to extract GM volumetric changes between two study groups. *P* value less than 0.001 combined with cluster size larger than 54 voxels was considered as statistically significant (false discovery rate (FDR) corrected). Results were visualized by the REST Slice viewer (rs-fMRI data analysis toolkit; http://www.restfmri.net/) [39].

Based on the two-sample *t*-test results, clusters of significant differences between the two groups were identified and further defined as ROIs. The volume of those ROIs was measured and recorded for each patient. Pearson's correlation analysis was performed using SPSS 17 software (SPSS, Inc., Chicago, IL) between the THI score, PT duration, and the volume of each ROI.

## 3. Results

**Figure 1:**
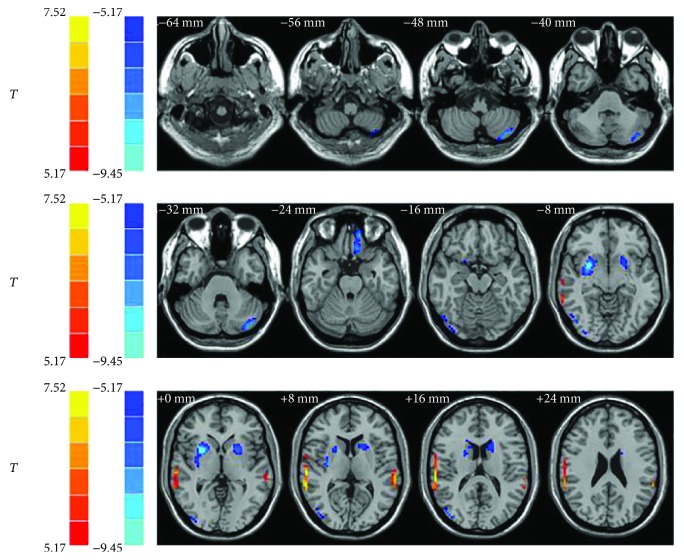
Result of volumetric changes between two groups. Compared with the normal controls, bilateral superior temporal gyrus showed significantly increased volumes among the pulsatile tinnitus patients (hot color). The left cerebellum posterior lobe, left frontal superior orbital lobe (gyrus rectus), right MOG, and bilateral putamen showed significantly decreased volumes (cold color). The left side corresponds to the right hemisphere. MOG = middle occipital gyrus.

In the whole brain analyses, a two-sample two-tailed *t*-test was performed to detect the differences between two groups of study subjects. As showed in Figure 1 and Table 2, we detected 7 clusters (regions of interest) with significant volume changes. Compared with the normal controls, bilateral superior temporal gyrus showed significantly increased volumes among the pulsatile tinnitus patients. However, the left cerebellum posterior lobe, left frontal superior orbital lobe (gyrus rectus), right middle occipital gyrus (MOG), and bilateral putamen showed significantly decreased volumes.

## 4. Discussion

There has been an increased interest in the pulsatile tinnitus-related research [14, 26–31, 34, 40–43]. In those neurofunctional studies, GM volumetric effects have been taken into account when investigating the regional neural activity changes. However, lower-dimensional computational methods in the SPM applied in spatial normalization step might be one of the reasons to explain why there was no significant GM volume difference reported between the PT patients and controls. In this study, DARTEL algorithm for image preprocessing in spatial normalization step was applied in order to acquire improved normalization results with higher resolution image sets that can be generated for image registration in the next step [44]. Smoothing parameters applied in the previous studies may also be one of the reasons. Smoothing can help ameliorate the effects of misalignment of anatomical structures when registration is less perfect. Different smoothing parameters could affect the possibility of neuroanatomical abnormality detections. Smoothing factor with an FWHM value twice of the resampled voxel size was recommended [45]. Thus, high-dimensional computational methods in the SPM with appropriate smoothing factor applied in this study are key factors in improving the possibility of neuroanatomical abnormality detections and ensuring the quality controls of this research.

The auditory cortex can be divided into primary auditory cortex (PAC), secondary auditory cortex (SAC), and auditory association cortex (AAC). Our results indicated increased GM volume in the bilateral auditory network (superior temporal gyrus), mainly overlapped with AAC. AAC is considered to have more important roles than PAC and SAC in the functional attribution of tinnitus [13]. As a relatively more sensitive region, abnormalities could mainly be found in the AAC rather than other parts of the auditory cortex in tinnitus patients [27, 46–49]. It was also supported by the bilaterally increased GM volume in the AAC, rather than the PAC or SAC, in this study. Those brain regions could be neuroanatomical features of the patients with pulsatile tinnitus in the early stage of PT symptom. What should be noticed is that the perception of pulsatile tinnitus is not a consequence of structural brain alterations. Also, the etiology of patients enrolled in this study was confirmed as SPD by CTA/V. Neuroanatomical changes would also not be considered as etiology of PT. Thus, we may only make primary hypothesis that those neuroanatomical findings might occur as a consequence of the longer lasting exposure to PT. Further studies are still needed to investigate the feature of alterations and its biological meaning of the AAC as well as the whole auditory cortex in PT patients.

Cerebellum posterior lobe is known to participate in auditory processing [50]. In the current study, only patients with consistent right-sided unilateral tinnitus were enrolled, and brain volume alterations were only found in the left cerebellum posterior lobe. Previous studies on auditory pathway demonstrated predominantly contralateral involvement to the side of sound stimulation [51, 52]. Similar changes were also reported in patients with unilateral NPT [37, 53–55]. The brain volume changes in the left cerebellum in patients with right-sided PT may be caused by contralateral effects of the auditory perception, which was not previously reported.

In this study, only unilateral right-sided PT patients were recruited; however, the GM volume was found to be bilaterally altered (e.g., alterations in the left and right superior temporal gyrus). This finding was also reported in previous functional studies of PT patients [27, 28]. This may indicate that the brain is working as a whole organ when processing auditory messages. The auditory cortexes in both the left and right hemisphere of the brain were all affected by PT. According to previous studies, the contralateral side of the brain mainly responses to the monoaural sensory input [51, 52, 56]. For tinnitus patients, the strong lateralization of cortical response was proved to be significantly decreased [53, 54, 57]. Thus, in theory, changes in the left side of the auditory cortex may be more significant than that on the right side. However, compared with the right side, the brain volume change on the left side has lower peak intensity with smaller affected areas. There were also studies indicating that the right-hemispheric is specialized for auditory rhyme processing [58]. In consideration of previous brain functional studies [27, 46, 49, 58], our study may provide another evidence to support the opinion that right-sided auditory cortex may also be more vulnerable than the left-sided one from the anatomical aspect. Further investigations toward both the left- and right-sided unilateral PT patients may provide direct evidence to support our hypothesis.

The putamen is a part of the basal ganglia. Regional and network functional deficits associated with putamen may reflect an imbalance in neuromodulation of auditory verbal hallucinations (AVHs) [59]. A neuroanatomical study on chronic tinnitus did not indicate altered brain volume and reduced functional connectivity in the putamen [60]. Brain volume changes in the bilateral putamen may be a specific finding for PT patients, and its clinical relevance still needs to be investigated.

Visual brain areas were reported to be involved in auditory spatial information processing [61–63]. It could be divided into dorsal and ventral visual areas. Compared with the ventral visual areas, the dorsal visual areas may experience greater neural plasticity in patients in the early stage of PT symptom [64, 65]. The MOG is part of the dorsal visual areas. Previous neurofunctional studies reported the involvement of MOG in patients with PT [27, 29], indicating reorganized functional activity after the perception of PT. In this study, neuroanatomical changes of MOG were also reported in the PT group compared with normal controls. It may due to the connections between the auditory network and visual network [66–69]. However, the correlation between the brain volume of MOG and THI score was not statistically significant, indicating that the brain volume of dorsal visual areas, especially MOG, would not be significantly affected in the early stage of pulsatile tinnitus symptom.

Combined with previous findings, we also found that anatomical and functional changes may not correlate to each other in patients with pulsatile tinnitus. In a previous rs-fMRI study [26], decreased amplitude of low-frequency fluctuation (ALFF) value was found in the bilateral cerebellum posterior lobe, representing decreased regional neural activity in bilateral brain areas. However, decreased brain volume was only found in the left cerebellum posterior lobe in this study. This suggested that anatomical and functional changes of the brain may not be consistent in PT patients. Previous studies showed no abnormal regional neural activity in the auditory cortex [28, 30], but there are alterations of functional connectivity between the right AAC and the default mode network in patients with right-sided unilateral PT [27]. Our neuroanatomical studies presented in this work indicated GM volumetric changes detected in the bilateral AAC for the same type of patients. These results also indicated possible noncorrelated changes between regional neural activity, functional connectivity, and GM volumes.

There are some limitations in this study. Firstly, only right-sided PT patients in the early stage of PT symptom were enrolled in this study. Studies including patients with left- and right-sided pulsatile tinnitus are still required in order to have a better understanding of the symptom. Secondly, only 24 PT patients were included in this study. A study with larger sample size will be conducted in our future neuroanatomical studies of PT patients.

## 5. Conclusions

This was the first study specifically which explored the brain anatomical alterations and demonstrated the features of neuroanatomical changes in patients with unilateral PT during their early stages of the symptom. From this study, a more reliable framework specifically for the study of anatomical abnormalities in the patients with unilateral PT has been generated, which would provide new insights for the brain changes to the patients with PT from a neuroanatomical perspective.

## Figures and Tables

**Table 1:** Characteristics of the participants.

	PT (*n* = 24)	HC (*n* = 24)	*P* value
Age (years)	24~53 (34.9 ± 7.9)	23~59 (35.3 ± 9.0)	0.906^a^
Gender (male/female)	3/21	3/21	1.000^b^
Handedness	24 right-handed	24 right-handed	1.000^a^
PT duration (months)	3~37 (17.9 ± 11.6)		
THI score	18~94 (47.8 ± 22.8)		

Data are presented as the ranges of Min–Max (means ± standard deviations). PT: pulsatile tinnitus; HC: healthy controls. ^a^Two-sample two-tailed *t*-test. ^b^Fisher's exact test.

**Table 2:** Regions showed a significant difference in volumes between PT patients and the controls (*P* < 0.001, FDR corrected).

Brain region	Peak MNI (mm)			Peak *T* value	Number of voxels
	*x*	*y*	*z*		
L cerebellum posterior lobe	−33	−78	−51	−8.1425	115
L superior orbital lobe (rectal gyrus)	−12	45	−27	−8.6597	70
R middle occipital gyrus	54	−69	−12	−7.4936	59
R putamen	27	6	0	−9.4527	262
L putamen	−21	9	3	−7.3238	146
R superior temporal gyrus	63	−27	15	7.5155	192
L superior temporal gyrus	−63	−30	9	7.0531	57

R: right; L: left; MNI: Montreal Neurological Institute. Correlation between volumes of ROI and duration of the pulsatile tinnitus. THI score was also analyzed. No significant correlation was found.
